# Balancing the maintenance strategies to making decisions using Monte Carlo method

**DOI:** 10.1016/j.mex.2024.102819

**Published:** 2024-06-29

**Authors:** Khamiss Cheikh, E. L. Mostapha Boudi, Rabi Rabi, Hamza Mokhliss

**Affiliations:** a Department of Mechanical Engineering, Energetic team,Mechanical and Industrial Systems (EMISys), Mohammadia School of Engineers, Mohammed V University, Rabat, Morocco; b Department of Physics (LPM-ERM), Faculty of Sciences and Techniques, Sultan Moulay Sliman University, B.P.523, Beni-Mellal 23000, Morocco; c Department of Physics, Laboratory of Electronics, Instrumentation and Energetics, Faculty of Sciences, Chouaib Doukkali University, El Jadida, Morocco

**Keywords:** Performance, Robustness, Condition-based maintenance, Periodic inspection, Quantile-based inspection, Renewal process, Monte Carlo method, Monte Carlo Method

## Abstract

This study aims to develop comprehensive maintenance strategies tailored to enhance the dependability, performance, and lifespan of critical assets within industrial and organizational settings. By integrating proactive, preventive, predictive, and corrective maintenance tactics, our strategy seeks to minimize downtime, reduce costs, and optimize asset performance. Drawing from extensive case studies across various industrial sectors, our research utilizes robust data analysis to inform strategy development.

We employ mathematical cost models and simulations using the Monte Carlo Method in MATLAB to evaluate the performance and robustness of different maintenance strategies, including time-based and condition-based approaches. Our findings demonstrate that a holistic maintenance approach significantly improves operational efficiency and asset longevity. Specifically, our analysis reveals that integrated maintenance strategies lead to reduced downtime, lower maintenance costs, and enhanced asset reliability.

Policy implications of our research suggest that organizations should adopt integrated maintenance strategies to enhance asset reliability and performance, ultimately achieving sustained operational excellence. By emphasizing the importance of proactive maintenance measures alongside traditional reactive approaches, organizations can effectively manage their critical assets, leading to improved operational outcomes and long-term success.

– Integration of proactive, preventive, predictive, and corrective maintenance tactics
– Evaluation of performance and robustness through mathematical cost models
– Application of the Monte Carlo Method in MATLAB for comparative analysis

## Graphical abstract

**Figure:**
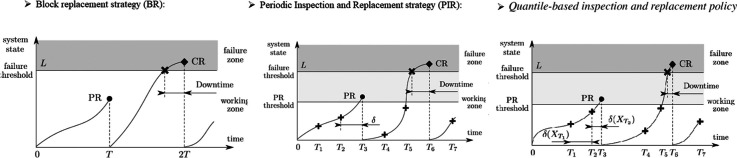

Specifications table

*This table provides general information on your method.*

**Table:** 

Subject area:	Asset Management and Maintenance Optimization in Industrial and Organizational Contexts
More specific subject area:	Comparative Analysis of Time-Based and Condition-Based Maintenance Strategies for Asset Optimization in Industrial Settings.
Name of your method:	Monte Carlo Method
Name and reference of original method:	The original method referred to in the article is the ``Monte Carlo Method.'' It is named after the Monte Carlo Casino in Monaco, known for its games of chance and randomness, as the method relies on random sampling techniques. The method was first introduced by physicists Stanislaw Ulam and John von Neumann in the 1940s as a way to solve problems in nuclear physics. **Reference:** Ulam, S., & von Neumann, J. (1949). ``On the Monte Carlo method.'' Los Alamos Scientific Laboratory, Report No. LA-1940.
Resource availability:	Not applicable

## Background

The motivation behind providing this methodology lies in the recognition of maintenance as a critical enabler of organizational success in today's fast-paced and competitive business environment. By adopting a proactive, comprehensive approach to maintenance, organizations can enhance asset reliability, minimize downtime, optimize costs, and ultimately achieve sustained operational excellence.

## Method details

The proposed methodology for maintenance plan development emphasizes a comprehensive approach encompassing proactive, preventive, predictive, and corrective strategies. This methodology aims to optimize asset performance, minimize downtime, and reduce costs in industrial and organizational settings.

## Method validation

Certainly, while a methods article typically focuses on detailing the methodology rather than presenting results or discussing them, providing validation data can indeed enhance the credibility and applicability of the proposed method. Here's an outline of how validation data could be presented: Consequently, in order to attain our aims, in both system types, Type I and Type II, The succeeding portions of this work are arranged as follows: In Section II, we briefly explore the deterioration and failure model. Section III provides the maintenance assumptions and cost models for the BR, PIR, and QIR techniques. Section IV thoroughly analyse the performance and robustness of the techniques. Detailed comparisons of the three strategies, BR, PIR and QIR for both system types, Type I and Type II, employing the new criteria, are described in Section V. Finally, we finish the work and provide some views in Section VI.

## Introduction

In today's competitive landscape, effective maintenance policies are crucial for organizations to maintain market competitiveness, ensure product/service quality, and meet client demands consistently [1]. However, the evolving nature of industries necessitates the development of new methodologies for quantitatively evaluating maintenance policies to gauge economic performance and resilience [2,3].

Recent research has highlighted the advantages of Condition-Based Maintenance (CBM) strategies over Time-Based Maintenance (TBM) strategies, particularly in systems with variable degradation processes [[4], [5], [6]]. CBM's adaptability, driven by continuous system monitoring, yields cost-saving benefits compared to the rigid nature of TBM. Nonetheless, TBM may prove economically favorable in scenarios of stable system behavior [6].

Moreover, considering cost variability within maintenance approaches can impact the long-term maintenance cost rate $C_{\infty }$, potentially affecting system performance [6]. These insights prompt critical questions regarding the extent of CBM's economic advantage, the performance of maintenance techniques across different system characteristics, the possibility of reducing maintenance costs without compromising performance, and the variables influencing maintenance strategy resilience.

To address these questions, our research aims to conduct a comprehensive evaluation of maintenance procedures, analyzing performance and robustness, and identifying maintenance technique performance across various system characteristics and their implications for economic performance and resilience. By filling existing literature gaps and offering practical recommendations to inform maintenance management decision-making, we aim to contribute to a deeper understanding of maintenance management and its implications for organizational and economic resilience.

To achieve our objectives across both system types, Type I and Type II, the subsequent sections of this paper are organized as follows: Section II briefly examines the deterioration and failure model. Section III presents the maintenance assumptions and cost models for the BR, PIR, and QIR techniques. Section IV conducts a comprehensive analysis of the performance and robustness of these techniques. In Section V, detailed comparisons of the three strategies, BR, PIR, and QIR, for both system types are provided, employing the new criteria. Finally, Section VI concludes the work and offers insights for future research.

## Degradation and failure model

To accurately represent the degradation of a system, we introduce the scalar random variable *X*_t_. This variable quantifies the level of degradation at any given time *t* ≥ 0. It serves as a precise measure of the deterioration experienced by the system throughout its operational lifespan. The sequence {X_t_} represents the degradation in the absence of maintenance interventions. The stochastic process *t*≥0 is increasing and commences from an initial value of *X*_0_ = 0, representing the system's pristine state.

To model the system's degradation, we assume that the degradation increment between two time points, *t* and *s* (where *t* ≤ *s*), denoted as *Xs* − *X_t_*, is statistically independent of the degradation levels observed prior to *t*. This assumption suggests that the future degradation increment is solely dependent on the current degradation level and the time interval considered, without being influenced by the historical degradation pattern. By incorporating this assumption, we can accurately represent the system's degradation behaviour and analyse its progression over time.

To model the system's degradation progression, we can use various monotonic stochastic processes from the Lévy family [7]. The Lévy family comprises a diverse range of mathematical models that exhibit monotonicity. These models provide a flexible and versatile framework to capture the trajectory of the system's degradation. They allow us to simulate and analyze how the system's degradation evolves over time while accounting for its unique characteristics and the inherent variability present in the degradation process.

By selecting an appropriate process from the Lévy family, we can examine the system's degradation progression in detail. These processes reflect the specific degradation patterns observed in the system under study, taking into account factors such as wear and tear, aging, or other forms of deterioration. The choice of the specific Lévy process depends on the particular characteristics and behaviours exhibited by the system being analysed.

The paper selects the homogeneous Gamma process, which is a well-established stochastic process, to model the phenomenon of degradation. The Gamma process is characterized by two parameters: the shape parameter (*α*) and the scale parameter (*ß*).

The decision to use the Gamma process is supported by a range of practical applications in different fields. The paper cites specific examples such as corrosion damage mechanisms [8], degradation of carbon-film resistors [9], SiC MOSFET threshold voltage degradation [10], fatigue crack propagation [11], and performance loss in actuators [12]. These applications demonstrate that the Gamma process has been successfully used to model degradation phenomena in various contexts.

Additionally, the choice of the Gamma process is further validated by expert opinions [13]. Experts in the field endorse the suitability of the Gamma process for modelling degradation, adding credibility to its selection in the paper.

One of the advantages of using the Gamma process is its mathematical formulation. The Gamma process offers mathematical tractability, which means it provides analytical solutions and convenient mathematical properties. This tractability facilitates the analysis and interpretation of the degradation process. Researchers can gain insights into the behaviour of the system over time by utilizing the mathematical properties of the Gamma process.

In the context of the paper, for the time interval *t* ≤ *s*, the degradation increment *X_s_* − *X_t_* follows a Gamma distribution with a probability density function. This implies that the degradation increment can be modelled probabilistically, and the specific form of the Gamma distribution allows for quantifying the degradation increment within the given time interval. Therefore, for *t* ≤ *s*, the increase in degradation *X_s_* − *X_t_* follows a Gamma distribution with a probability density function:

(1) $$f_{\alpha .\left(s-t\right),\beta }\left(x\right)=\frac{\beta^{\alpha .\left(s-t\right)}x^{\alpha .\left(s-t\right)-1}e^{-\beta x}}{\Gamma \left(\alpha .\left(s-t\right)\right)}.1_{\left\{x\geq 0\right\}},$$

And survival function:

(2) $${\bar{F}}_{\alpha .\left(s-t\right),\beta }\left(x\right)=\frac{\Gamma \left(\alpha .\left(s-t\right),\;\beta x\right)}{\Gamma \left(\alpha .\left(s-t\right)\right)},$$

Where $\Gamma \left(\alpha \right)=\int_{0}^{\infty }z^{\alpha -1}e^{-z}dz$ and $\Gamma \left(\alpha ,x\right)=\int_{x}^{\infty }z^{\alpha -1}e^{-z}dz$ represent the complete and upper incomplete Gamma functions, respectively.

Additionally, let's delve further into the indicator function, denoted as 1{⋅}, which serves as a powerful tool for expressing conditions within our mathematical framework. This function allows us to effectively delineate between true and false conditions, providing a clear binary output that aids in the precise formulation of our models. By leveraging the indicator function, we can seamlessly incorporate logical constraints and decision criteria into our analysis, enhancing the robustness and interpretability of our results.

Expanding on the parameters (α, *ß*), we recognize their significance in capturing the diverse range of degradation behaviours exhibited by complex systems. These parameters offer a flexible means of calibrating our models to accurately reflect the underlying dynamics of degradation processes. By adjusting the values of α and *ß*, we can modulate the average degradation rate and variance, thereby accommodating different levels of uncertainty and variability in system behaviour. This versatility enables us to tailor our models to specific contexts and scenarios, facilitating a more nuanced understanding of system performance and reliability.

Central to our analysis are the concepts of average degradation rate and variance, which provide key insights into the temporal evolution and variability of system degradation. The average degradation rate, expressed as m=α/β, quantifies the rate at which system performance deteriorates over time. This metric serves as a fundamental indicator of system health, with higher values indicating more rapid degradation and lower values suggesting more gradual deterioration. Similarly, the variance, denoted by $\text{var}=\alpha /\beta^{2}$, measures the dispersion or spread of degradation rates around the mean. A higher variance implies greater variability in degradation rates, reflecting fluctuations in system performance, while a lower variance indicates more consistent behavior.

Turning to the practical application of our models, we distinguish between two distinct types of systems based on their degradation characteristics:

• Type I systems, characterized by parameters *α*_1_ = 0.1, *ß*_1_ = 0.1, exhibit variable behavior. This configuration results in an average degradation rate to m_1_ =1 and a variance equal to var_1_ = 10, capturing the inherent variability in such systems. Type I systems are often subject to unpredictable environmental factors or operating conditions, leading to fluctuating degradation rates over time.
• Type II systems, defined by parameters *α*_2_ = 0.5, *ß*_2_ = 0.5, demonstrate more stable behavior. This configuration yields an average degradation rate m_2_ = 1 and a variance equal to var_2_ = 2, reflecting a more consistent degradation pattern. Type II systems typically exhibit less variability in degradation rates, making them more predictable and easier to manage from a maintenance perspective.

In the context of system degradation, we adopt a threshold-type model to define failure. Indicators for this model would likely revolve around monitoring parameters that signify the progression of degradation towards the defined threshold. Jardine, Lin, and Banjevic (2006) provide a comprehensive review on machinery diagnostics and prognostics implementing condition-based maintenance, highlighting the importance of various indicators in predicting failures, such as performance metrics and health indices [14].

Barabadi and Golestaneh (2017) discuss condition-based maintenance in industrial applications, emphasizing the role of health indices and performance metrics in defining maintenance strategies. They illustrate how these indicators can be used to monitor system degradation and identify when a system is approaching its failure threshold [15].

Li and Lee [16] focus on prognostics and health management design for rotary machinery systems, offering insights into predictive maintenance signals and time-to-failure estimates. They explain how early warning signs or patterns in data can precede system failure, making them crucial for threshold-type models that rely on accurately predicting when a system will reach its failure point.

This model considers a range of factors, including economic considerations such as product quality and resource utilization, as well as safety concerns such as the risk of hazardous breakdowns. A system is deemed to have failed when it can no longer fulfill its intended purpose in an acceptable condition, regardless of its technical operability. This definition of failure takes into account both functional and non-functional aspects of system performance, emphasizing the importance of maintaining system integrity and reliability.

Within this framework, we posit that system failure occurs once the degradation level surpasses a pre-established critical threshold L. This threshold represents a tipping point beyond which the system's performance becomes unacceptable, necessitating intervention to prevent further deterioration and mitigate associated risks. By proactively monitoring degradation levels and implementing timely maintenance interventions, organizations can effectively manage the reliability and availability of critical systems, ensuring their continued functionality and performance.

In this context, let $\tau_{L}$ represent the random failure time of the system, which can be expressed as:

(3) $$\tau_{L}=\text{inf}\;\left\{t\in R^{+}|X_{t}\geq L\right\}$$

The density function of $\tau_{L}$ at time *t* ≥ 0 is given by [17]:

(4) $$f_{\tau L}\left(t\right)=\frac{\alpha }{\Gamma \left(\alpha t\right)}\int_{L\beta }^{\infty }\left(\ln\left(z\right)-\psi \left(\alpha t\right)\right)z^{\alpha t-1}e^{-z}dz,$$

Where $\psi \left(\nu \right)=\frac{\partial }{\partial \nu }\ln\left(\Gamma (\nu )\right)$ is known as digamma function.

## Maintenance strategies and cost models

In this part, we outline the specific assumptions related to the system being maintained. These assumptions can include factors such as the system's operational environment, expected usage patterns, failure modes, component lifetimes, and any other relevant characteristics that influence the choice of maintenance strategies. These assumptions ensure that our analysis aligns with the specific requirements and constraints of the system under consideration.

After establishing the assumptions, we provide comprehensive decision criteria for each maintenance strategy. For the block replacement (BR) strategy, decision criteria may include factors such as the system's reliability requirements, the anticipated degradation rate of components, the cost of replacement parts, and the desired level of system availability. For the Periodic Inspection and Replacement strategy (PIR) and quantile-based inspection and replacement strategy (QIR) as condition-based maintenance (CBM) approaches, decision criteria may encompass elements such as the probability of failure detection during inspections, the cost of inspections, the impact of failure on system performance, and the availability of replacement parts. These decision criteria help us evaluate the advantages and disadvantages of each strategy and make informed decisions regarding their suitability for the system.

Finaly, we will illustrate the process of formulating the Maintenance Cost per Renewal Cycle (MCPRC) for both System Type 1 and System Type 2. This calculation involves quantifying the costs associated with each maintenance strategy over a renewal cycle, including costs for inspections, replacements, system downtime, and any other relevant expenses. By calculating the MCPRC for the block replacement (BR) strategy, the Periodic Inspection and Replacement strategy (PIR), and the quantile-based inspection and replacement strategy (QIR) for both system types, we can compare their economic feasibility and determine the most cost-effective approach.

### Maintenance assumptions

The system offers two maintenance options: Preventive Replacement (PR) and Corrective Replacement (CR). PR involves scheduled replacements or repairs to prevent failures, while CR addresses unexpected failures. CR typically incurs higher costs due to its unplanned nature and potential environmental impact [18]. Additionally, maintenance costs vary based on the system's degradation level, with more deteriorated systems requiring more complex and expensive repairs. PR and CR costs increase with system degradation, following the relationship 0 < C_i_ < C_p_(X_t_) < C_c_(X_t_), where C_i_ represents inspection costs. Replacements can only occur at specific times, leading to downtime after a failure, incurring additional costs until the next replacement opportunity. Decision-makers must carefully evaluate these factors to optimize maintenance strategies for system reliability and cost-effectiveness.

Where:C_p_ (X_t_) and C_c_ (X_t_) are often used to represent specific types of costs associated with maintenance activities, where X_t_ typically denotes the state of the system or equipment at time t. Here are the definitions for each:

• C_p_ (X_t_) - Preventive Maintenance Cost**:** represents the cost of performing preventive maintenance when the system is in state X_t_. Preventive maintenance is carried out to prevent failures and typically includes regular inspections, adjustments, replacements of parts, and other actions intended to maintain the equipment's reliability and extend its useful life. The cost C_p_ (X_t_) can depend on the state X_t_ ​, which might include factors such as the age of the equipment, usage patterns, and wear levels.
• C_c_ (X_t_) - Corrective Maintenance Cost: denotes the cost of corrective maintenance (or repair) when the system is in state X_t_ ​. Corrective maintenance is performed after a failure has occurred and includes actions taken to restore the system to operational condition. The cost C_c_ (X_t_) can vary based on the severity of the failure, the state of the system at the time of failure, the time required to repair, the cost of replacement parts, and the impact on production or operations.

### Maintenance strategies

(1) Block replacement strategy (BR):

This scenario exemplifies Time-Based Maintenance (TBM) strategies, where decision-making hinges upon a straightforward framework predominantly reliant on calendar time blocks. In this approach, the system undergoes replacement at regular intervals denoted as kT, where K takes on integer values starting from 1, incrementing sequentially (i.e., K=1, 2, ...).

Maintenance actions are executed either proactively if the system remains operational at the specified replacement time (i.e., X_kT_ < L), or reactively if the system experiences a malfunction or significant degradation (i.e., X_kT_ ≥ L).

The replacement period T plays a pivotal role in determining the strategy, with each T interval delineating the timing of replacements. Consequently, the Basic Replacement (BR) strategy is dictated by this predetermined time-based framework, offering a structured approach to maintenance scheduling and execution.

(2) Periodic inspection and replacement strategy (PIR):

The Preventive Inspection and Replacement (PIR) approach is a basic tactic within Condition-Based Maintenance (CBM). It involves the following steps:

• Periodic Inspections: Regular inspections are conducted at defined intervals (δ) between inspections, denoted as Tk = kδ, where k represents the inspection number.
• Deterioration Level Assessment: At each inspection time (Tk), the system's deterioration level (XTk) is assessed based on collected indicators and measurements during the inspection.
• Decision-making: Based on the observed deterioration level (XTk), a decision is made regarding the maintenance action:
  a. Preventive Replacement (PR): If the deterioration level exceeds a predefined threshold or reaches a critical state, a proactive replacement is performed to prevent potential failures or degradation.
  b. Corrective Replacement (CR): If the deterioration level is below the threshold or not critical, no immediate replacement is needed. The system continues operating until the next inspection.

The PIR approach maintains a constant inspection period while linking inspection times to preventive and corrective replacement activities. Its goal is to optimize maintenance actions and manage system reliability and performance effectively.

(3) Quantile-based inspection and replacement strategy (QIR):

Using a quantile schedule specified by the parameter α, where 0 < α < 1, the Condition-Based Inspection and Replacement (QIR) technique assesses the system in contrast to the Periodic Inspection and Replacement (PIR) method. This indicates that, in comparison to PIR, QIR uses a more dynamic and adaptable approach. Rather of depending on predetermined intervals for inspections, QIR modifies its inspection schedule according to the quantile α, which is a percentile of the degradation distribution of the system.

(5) $$T_{k+1}=T_{k}+\Delta T_{k+1},\;\Delta T_{k+1}=\delta \left(X_{T_{k}}\right)=\text{inf}\left\{t\geq 0,R(t|\;X_{T_{k}})\geq \alpha \right\},\;k=1,\;2,\;...$$

The conditional reliability of the system at time t is given by R(t | $X_{T_{k}}=x_{k}$), X_T0_ = X_0_ = 0, and the system's degradation level at the inspection time T_k_, denoted by X_k_. The following may be done to ascertain this conditional dependability using $X_{T_{k}}=x_{k}$:

(6) $$R\left(t\;/\;X_{Tk}=x_{0}\right)=\;1-\;{\bar{F}}_{\alpha .\left(t-T_{k}\right),b}\left(L-x_{k}\right),$$

where ${\bar{F}}_{\alpha .(t-T_{k}),b}\left(L-x_{k}\right)\;$is given by (2). According to Eq. (5), the system depends on at least α throughout the inspection period [T_k_, T_k+1_]. Stated differently, the quantile-based inspection technique makes sure that α is the minimal reliability level for the duration of the system's life.

### Maintenance cost per renewal cycle

As previously said, we are in favor of evaluating the reliability of the BR, PIR, and QIR approaches using the Maintenance Cost per Renewal Cycle (MCPRC). The length of a renewal cycle, *S*, and the total maintenance cost incurred throughout the cycle, (*S*), are the two parameters that characterize the MCPRC.

(7) $$K=\frac{C\left(S\right)}{S}.$$

Since *K* is a random variable, we try to evaluate it using *µ* = *E* (*K*), which is the mean value and standard deviation.

(8) $$\sigma =\sqrt{E\left(K^{2}\right)-E^{2}\left(K\right)}=\sqrt{E\left(K^{2}\right)-\mu^{2}}.$$

It seems that the robustness of the maintenance processes declines as the standard deviation (*τ*) values rise. The analytical formulas for σ for the two methodologies that are being considered are delineated in the subsequent sections.

1) Standard formulation of the MCPRC for the BR strategy:

The chance of failure during a renewal cycle, the expected number of failures, preventative replacements, and the overall maintenance cost incurred are used to determine the MCPRC (Maintenance Cost per Renewal Cycle) of the BR (Block Replacement) plan. This covers the price of maintaining equipment through corrective repairs and preventative replacements, as well as the expense of equipment malfunction-related downtime. By dividing the entire maintenance cost by the duration of the renewal cycle, the MCPRC is computed. The BR approach offers an average measurement of the maintenance cost per unit of time and is comparably cost-effective to other maintenance methods.

For this reason, the MCPRC of the BR strategy may be calculated as follows:

(9) $$K^{BR}=\left(C_{p}\left(X_{T}\right)\cdot 1\left\{X_{T}<L\right\}+C_{C}\left(X_{T}\right)\cdot 1\left\{X_{T}\geq L\right\}+C_{d}W_{d,BR}\right)/T,$$

In this case, *W_d_*_,BR_ is the system outage that occurs during a renewal cycle in accord with the BR approach. We can describe it as:

(10) $$W_{d,BR}=\left(T\;-\;\tau_{L}\right)\cdot 1_{\left\{\tau_{L}<T\right\}}=\;\int_{0}^{T}1_{\left\{X_{t}<L\right\}}dt,$$

The computation of *µ^BR^* and σ*^BR^* uses both iterations of *W_d_*_,BR_. Consequently, the expressions of the mean *µ^BR^* =[*K^BR^*] and mean of square E[(*K*^BR^)²] of MCPRC are:

(11) $$\mu^{BR}=\left(\int_{0}^{L}C_{p}\;\left(x\right)\;f_{\alpha T,\beta }\;\left(x\right)\;dx+\int_{L}^{\infty }C_{C}\;\left(x\right)\;f_{\alpha T,\beta }\;\left(x\right)\;dx\;+C_{d}\int_{0}^{T}{\bar{F}}_{\alpha t,\beta }\;\left(L\right)\;dt\right)/T,$$

And

(12) $$\begin{array}{ccc} E\left[{\left(K^{BR}\right)}^{2}\right] & = & \left(\int_{0}^{L}{C_{p}}^{2}\;\left(x\right)\;f_{\alpha T,\beta }\;\left(x\right)\;dx+\int_{L}^{\infty }{C_{c}}^{2}\;\left(x\right)\;f_{\alpha T,\beta }\;\left(x\right)\;dx\;+\;2C_{d}\int_{0}^{T}(\int_{L}^{\infty }(\int_{x}^{\infty }C_{p}\;\left(z\right)\;f_{\alpha T,\beta }\;\left(z\right)\;dz\;\right)\;f_{\alpha t,\beta }\;\left(x\right)\;dx)dt\\ & & +\;{C_{d}}^{2}\;\int_{0}^{T}{\left(T-t\right)}^{2}\;f_{\tau_{L}}\;\left(t\right)\;dt)\;/\;T^{2}, \end{array}$$

In this case, $f_{\alpha T,\beta },\;{\bar{F}}_{\alpha t,\beta }$, and $f_{\tau L}\;$are respectively yielded by formulas (1), (2), and (4). By changing (11) and (12) to (8), we can obtain the formula for the standard deviation σ*^BR^* of the MCPRC within the framework of the BR strategy.

2) Standard formulation of the MCPRC for the PIR strategy:

Assume that, under the PIR (preventive Inspection and Replacement) strategy, the system is replaced either preventatively or correctively at the k-th inspection time, where k = 1, 2,..., within a renewal cycle of duration *S_k_* = *k*Ώ*T*. In this instance, the interval between inspections is denoted by Δ*T*.

During the renewal cycle, the system incurs costs for inspections, corrective replacement (CR), preventative replacement (PR), and downtime. The status of the system at the end of the cycle dictates the specific costs incurred.

The likelihoods of each potential event are taken into consideration when calculating the Mean Cost Per Renewal Cycle (MCPRC) of the PIR policy. An official statement from the MCPRC is as follows:

(13) $$K^{PIR}=\sum_{k=1}^{\infty }\left(\frac{C_{p}\;\left(X_{S_{k}}\right)+kC_{i}}{S_{k}}1_{\left\{X_{S_{k-1}}<M\leq X_{S_{k}}<L\right\}}+\;\frac{C_{c}\;\left(X_{S_{k}}\right)+kC_{i}}{S_{k}}1_{\left\{X_{S_{k-1}}<M<L\leq X_{S_{k}}\right\}}\;+\frac{C_{d}W_{d,PIR}\;}{S_{k}}\right),$$

Here, the PIR strategy's *W_d_*_,PIR_ denotes the system outage that happens inside the interval [*S_k_*_−1_, *S_k_*] (i.e., during a renewal cycle). It is said as follows:

(14) $$W_{d,PIR}=\left(S_{k}-\tau_{L}\right).\;1_{\begin{array}{c} \left\{S_{K-1}<\tau_{L}\leq S_{K}\right\}\\ \; \end{array}}\;=\;\int_{S_{K-1}}^{S_{K}}\;1_{\left\{\;X_{S_{k-1}}<M<L\leq X_{t}\right\}}\;dt$$

Thus, the PIR strategy's mean MCPRC, *µ^PIR^* = [*K^PIR^*], is calculated as:

(15) $$\begin{array}{ccc} \mu^{PIR} & = & \sum_{k=1}^{\infty }\frac{1\;}{S_{k}}\int_{0}^{M}(\;\int_{M-x}^{L-x}\left(C_{p}\;\left(x+z\right)+k\;C_{i}\right)\times \;f_{\alpha \Delta T,\beta }\;\left(z\right)dz+\int_{L-x}^{\infty }\left(C_{c}\;\left(x+z\right)+k\;C_{i}\right)\;f_{\alpha \Delta T,\beta }\;\left(z\right)\;dz\\ & & +\;C_{d}\int_{S_{k-1}}^{S_{k}}{\bar{F}}_{\alpha \left(t-S_{k-1}\right),\beta }\;\left(L-x\right)\;dt)\;f_{\alpha S_{k-1},\beta }\;\left(x\right)dx \end{array}$$

where (1) and (2), respectively, provide $f_{\alpha (.),\beta }$ and ${\bar{F}}_{\alpha (.),\beta }$.The square root of *E*[(*K^PIR^*)^2^] related mean is as follows:

(16) $$\begin{array}{ccc} E\left[{\left(K^{PIR}\right)}^{2}\right] & = & \sum_{k=1}^{\infty }\{\int_{0}^{M}(\;\int_{\;M-x}^{\;L-x}\;{\left(C_{p}\;\left(x+z\right)+k\;C_{i}\right)}^{2}\times \;f_{\alpha \Delta T,\beta }\;\left(z\right)dz+\int_{L-x}^{\infty }\;{\left(C_{c}\;\left(x+z\right)+k\;C_{i}\right)}^{2}\\ & & \times \;f_{\alpha \Delta T,\beta }\;\left(z\right)\;dz)\;f_{\alpha S_{k-1},\beta }\;\left(x\right)dx\;+2C_{d}\int_{S_{k-1}}^{S_{k}}\;(\;\int_{0}^{M}(\;\int_{L-x}^{\infty }\left(\int_{x+z}^{\infty }\left(C_{c}\;\left(y\right)+k\;C_{i}\right)\;f_{\alpha S_{k},\beta }\;\left(y\right)dy\right)\\ & & f_{\alpha .\left(t-S_{k-1}\right),\beta }\;\left(z\right)dz)\;f_{\alpha S_{k-1},\beta }\;\left(x\right)dx\;)\;dt+{C_{d}}^{2}\;\int_{S_{k-1}}^{S_{k}}\;\;{\left(S_{k}-t\right)}^{2}\;\;f_{\tau_{L}}\;\left(t\right)\;dt\}\;/S_{k}, \end{array}$$

Here, _(⋅),_ and *f_τL_* are obtained by using Eqs. (1) and (4). Through integration of (15) and (16) into (8), we get the formula for the standard deviation σ*^PIR^* of the MCPRC using the PIR technique.

3) Standard formulation of the MCPRC for the QIR strategy:

There is a similarity between the Mean Cost Per Replacement Cycle (MCPRC) standard deviation for the QIR policy and the calculation for the PIR policy. Again, we assume that either a preventative or corrective system modification occurs during the kth inspection period (k = 1, 2,....). The QIR policy's MCPRC across a replacement cycle can be expressed as follows:

(17) $$\begin{array}{ccc} K^{QIR} & = & \frac{1}{\sum_{k=1}^{\infty }T_{k}.1_{\left\{X_{T_{k-1}}<M\leq X_{T_{k}}\right\}}}.\sum_{k=1}^{\infty }(\left(C_{p}\left(X_{T_{k}}\right)+kC_{i}\right).1_{\left\{X_{T_{k-1}}<M\leq X_{T_{k}}<L\right\}}\\ & & +(C_{c}\left(X_{T_{k}}\right)+kC_{i}).1_{\left\{X_{T_{k-1}}<M<L\leq X_{T_{k}}\right\}}+C_{d}W_{d,QIR}), \end{array}$$

where the QIR policy's downtime of the system during a renewal cycle is acquired by:

(18) $$W_{d,QIR}=\left(T_{k}-\tau_{L}\right).\;1_{\begin{array}{c} \left\{T_{K-1}<\tau_{L}\leq T_{K}\right\}\\ \; \end{array}}=\int_{T_{K-1}}^{T_{K}}\;1_{\left\{X_{T_{k-1}}<M<L\leq X_{t}\right\}}\;dt$$

and (5) determines T_k_ via a recursive process. The dynamic inspection schedule clearly makes it very challenging to compute µ^QIR^ = *E*[*K*^QIR^] and E[(*K*^QIR^)^2^] analytically in (17). Thus, we focus on calculating the standard deviation σ^QIR^ of the mean cost per replacement cycle (MCPRC) for the QIR policy using a Monte Carlo simulation approach.

## Maintenance strategies assessment

The long-term estimated maintenance cost rate criteria has been widely used in the literature [19] to assess the techniques' success. This criteria, which makes use of the classical renewal-reward theorem, may be expressed mathematically as [20]:

(19) $$C_{\infty }=\lim_{t\to \infty }\frac{E\left[C\left(t\right)\right]}{t}=\frac{E\left[C\left(S\right)\right]}{E\left[S\right]}$$

Where *S* is the period of a renewal cycle, and (*S*) is the cumulative maintenance cost spent throughout this cycle. Clearly, Eq. (19) only evaluates the mean values of the renewal cycle and its related maintenance cost, without taking into consideration the fluctuation in maintenance costs from one cycle to the next. In other words, focusing exclusively on long-term predicted maintenance cost rates may be insufficient for evaluating maintenance solutions in terms of performance and robustness. To overcome this constraint, we recommend using a cost criteria that combines the long-term projected maintenance cost rate ($C_{\infty }$) and the standard deviation of the MCPRC (σ). This may be phrased as:

(20) $$\varphi =C_{\infty }+\lambda .\sigma ;\;\lambda \geq 0.$$

Section III-C contains the mathematical formulae for σ under the BR, PIR, and QIR techniques.

Let's use a system characterized by the PR cost and modeled as a quadratic function of the degradation level to demonstrate the benefits of the new criterion. This cost of PR can be stated as follows:

(21) $$C_{p}\left(X_{t}\right)=C_{0}+\frac{C_{c}+C_{0}}{2}{\left(\frac{X_{t}-M_{s}}{L-M_{s}}\right)}^{2}\;1_{\left\{M_{s}<X_{t}<L\right\}},$$

Where L is the critical threshold, $C_{c}$ is the CR cost, and $C_{0}$ is the basic cost of PR. Additionally, M_s_ denotes a PR threshold.

We examine a stochastically degrading production system with parameters a = 0.1, b = 0.1, and L = 29 in this section. A thorough analysis is made possible by the selected values for the inspection cost (C_i_ = 5) and system downtime cost rate (C_d_ = 34). The landscape of our study is further defined by the CR cost (C_c_ = 98), basic PR cost (C_0_ = 48), and system degradation threshold (M_s_ = 14). Eq. (21) provides the basis for a rigorous mathematical analysis and is the fundamental framework for the PR cost.

This section aims to accomplish two main goals: firstly, it will provide numerical examples to verify the existence of optimal solutions for BR, PIR, and QIR strategies using the novel φ criterion, and secondly, it will highlight the significance of this criterion in achieving a balanced approach in contrast to the traditional C_∞_ criterion in maintenance strategies.

We apply the existing system and maintenance cost configuration described in (19) and (20) to achieve these objectives. To achieve the first goal, we use a Monte Carlo simulation technique with 2 500 histories to determine the best φ configurations for the BR, PIR, and QIR techniques. Setting the weight λ to 1.4 produces cost function shapes that, when painstakingly shown in Table 1, provide empirical evidence in favor of the existence of ideal configurations for various maintenance approaches.

**Table 1:** Optimal configurations φ of the BR, PIR, and QIR strategies.

Strategies	Relative weight	Optimal decision variables	Optimal configurations of φ
**BR**	λ=1.4	$T_{opt}=9.70$	$\varphi_{opt}^{BR}=11.738$
**PIR**	λ=1.4	$\Delta T_{opt}=6.19$ $M_{opt}=12.8$	$\varphi_{opt}^{PIR}=9.864$
**QIR**	λ=1.4	$\alpha_{opt}=0.55$ $M_{opt}=18.3$	$\varphi_{opt}^{QIR}=9.628$

**Fig. 1:**
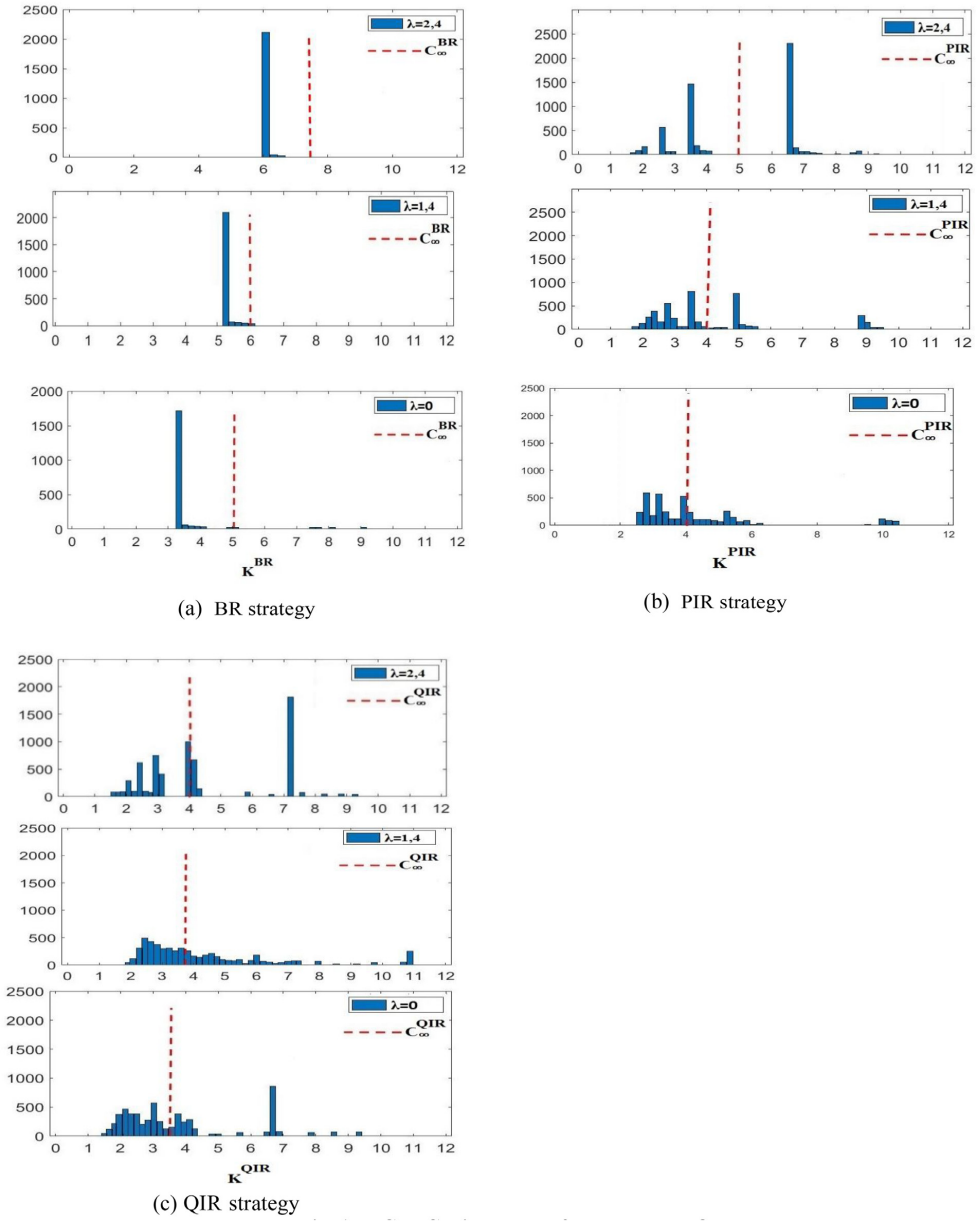
MCPRC Histogram of BR,PIR and QIR strategies.

Moving on to the second goal, the careful examination of the performance-robustness relationship in the BR, PIR, and QIR techniques is done. We analyze the long-term predicted maintenance cost rate C_∞_ and the histograms associated with their best configurations in Fig. 1 after optimizing these techniques based on the established criterion.

Table 2 delves deeply into the effects of different λ values on the standard deviation of MCPRC σ. Smaller MCPRC σ standard deviations are correlated with higher λ values, indicating more resilience attained through cost variability optimization. The complex trade-off between robustness and performance is highlighted by the fact that this increased resilience also raises the long-term estimated maintenance expense rate.

**Table 2:** Maintenance strategies optimization of the BR, PIR, and QIR strategies.

Strategies	Relative weight	Optimal decision variables	Long-run expected cost rate	Standard deviation of MCPRC
**BR**	λ=2.4	$T_{opt}=8.20$	$C_{\infty }^{BR}=6.713$	$\sigma_{\infty }^{BR}=3.721$
	λ=1.4	$T_{opt}=9.70$	$C_{\infty }^{BR}=5.986$	$\sigma_{\infty }^{BR}=4.109$
	λ=0	$T_{opt}=15.90$	$C_{\infty }^{BR}=5.054$	$\sigma_{\infty }^{BR}=5.682$
**PIR**	λ=2.4	$\Delta T_{opt}=8.29$ $M_{opt}=4.80$	$C_{\infty }^{PIR}=4.982$	$\sigma_{\infty }^{PIR}=4.409$
	λ=1.4	$\Delta T_{opt}=6.19$ $M_{opt}=12.8$	$C_{\infty }^{PIR}=4.103$	$\sigma_{\infty }^{PIR}=4.115$
	λ=0	$\Delta T_{opt}=5.50$ $M_{opt}=16.50$	$C_{\infty }^{PIR}=4.025$	$\sigma_{\infty }^{PIR}=5.009$
**QIR**	λ=2.4	$\alpha_{opt}=0.37$ $M_{opt}=8.80$	$C_{\infty }^{PIR}=4.063$	$\sigma_{\infty }^{PIR}=4.020$
	λ=1.4	$\alpha_{opt}=0.55$ $M_{opt}=18.3$	$C_{\infty }^{PIR}=3.832$	$\sigma_{\infty }^{PIR}=4.140$
	λ=0	$\alpha_{opt}=0.34$ $M_{opt}=15.60$	$C_{\infty }^{PIR}=3.667$	$\sigma_{\infty }^{PIR}=4.487$

The PIR strategy is consistently shown to have larger MCPRC standard deviations but smaller long-run expected cost rates, making it the more efficient option overall. This is evident from a thorough comparison of long-run expected cost rates and MCPRC standard deviations for the BR, PIR, and QIR strategies.

The study's conclusion highlights the possible conflict between maintenance techniques' robustness and performance and emphasizes the need to find a delicate balance in order to allocate funds and plan effectively. Section V offers a more thorough study that should shed more light on this complex connection.

## Maintenance strategies comparisons

As mentioned before, these maintenance procedures are used to two different kinds of systems:

• Type I: a system with variable behavior.
• Type II: a system with more or less stable behavior.

As a result, we compare the performance of the BR, PIR, and QIR strategies in both System Type 1 and System Type 2 in this section. We also assess how robust these strategies are in various scenarios involving different maintenance cost configurations and values of the relative weight parameter λ. This study aids in our comprehension of the relative merits of different maintenance strategies for various goals or scenarios in Type I and Type II systems. We can determine what settings are most effective for these methods under various combinations of maintenance costs and system features by examining how the optimal decision variables of these techniques vary. In essence, it helps us make well-informed decisions on maintenance plans depending on our unique requirements and situation.

In this paper, we introduce a novel method and its implementation, accompanied by detailed steps and a step-by-step numerical example. To address this concern, we will enhance the methodological section of the paper by incorporating step-by-step descriptions and numerical examples to ensure clarity and facilitate reader understanding. Specifically, we will provide detailed explanations of the mathematical models, simulation techniques, and implementation steps used in our study.

Here is a succinct overview of the method presented in this paper:

• Mathematical Models: We will elucidate the underlying mathematical principles behind our maintenance cost models, including the assumptions and equations used to quantify maintenance costs for different strategies (BR, PIR, and QIR).
• Simulation Techniques: We will describe the Monte Carlo Method in MATLAB in detail, outlining the steps involved in setting up and running the simulations to evaluate the performance and robustness of maintenance strategies.
• Implementation Steps: We will provide a step-by-step guide on how to implement our methodology, including data preparation, model setup, parameter estimation, simulation execution, and result interpretation.
• Numerical Examples: To illustrate the application of our methodology, we will include numerical examples that walk readers through the process of conducting maintenance strategy comparisons using simulated data.

### Sensitivity to the maintenance costs

This study helps identify the factors that have the greatest impact on the performance and robustness objective for both System Type 1 and System Type 2, which may help mitigate their negative influence. It also gives us an idea of which maintenance strategies are best suited for a given performance and robustness objective. We use the PR cost function from Eq. (21) with C_0_ = 48, M_s_ = 14, L = 29, and *α* = *ß* (*α* = *ß* = 0.1 for the system type I and *α* = *ß* = 0.5 for the system type II). We set λ to 1.4 for each of the two systems and maintain the CR cost constant at C_c_ = 98. The two configurations for inspection and downtime costs that follow are then taken into consideration:

○ Variable Inspection Cost: C_i_ is a variable that ranges from 1 to 45 in steps of 1, and the downtime cost per unit of time is fixed at C_d_ = 19.
○ 2. Variable Downtime Cost per Unit of Time: The inspection cost is fixed at C_i_ = 7, while C_d_ swings between 10 and 50 with an increment of 1.

The maintenance expenses fulfill the criteria 0 < C_i_ < C_p_ (X_t_) < C_c_ (X_t_), it should be emphasized.

**Fig. 2:**
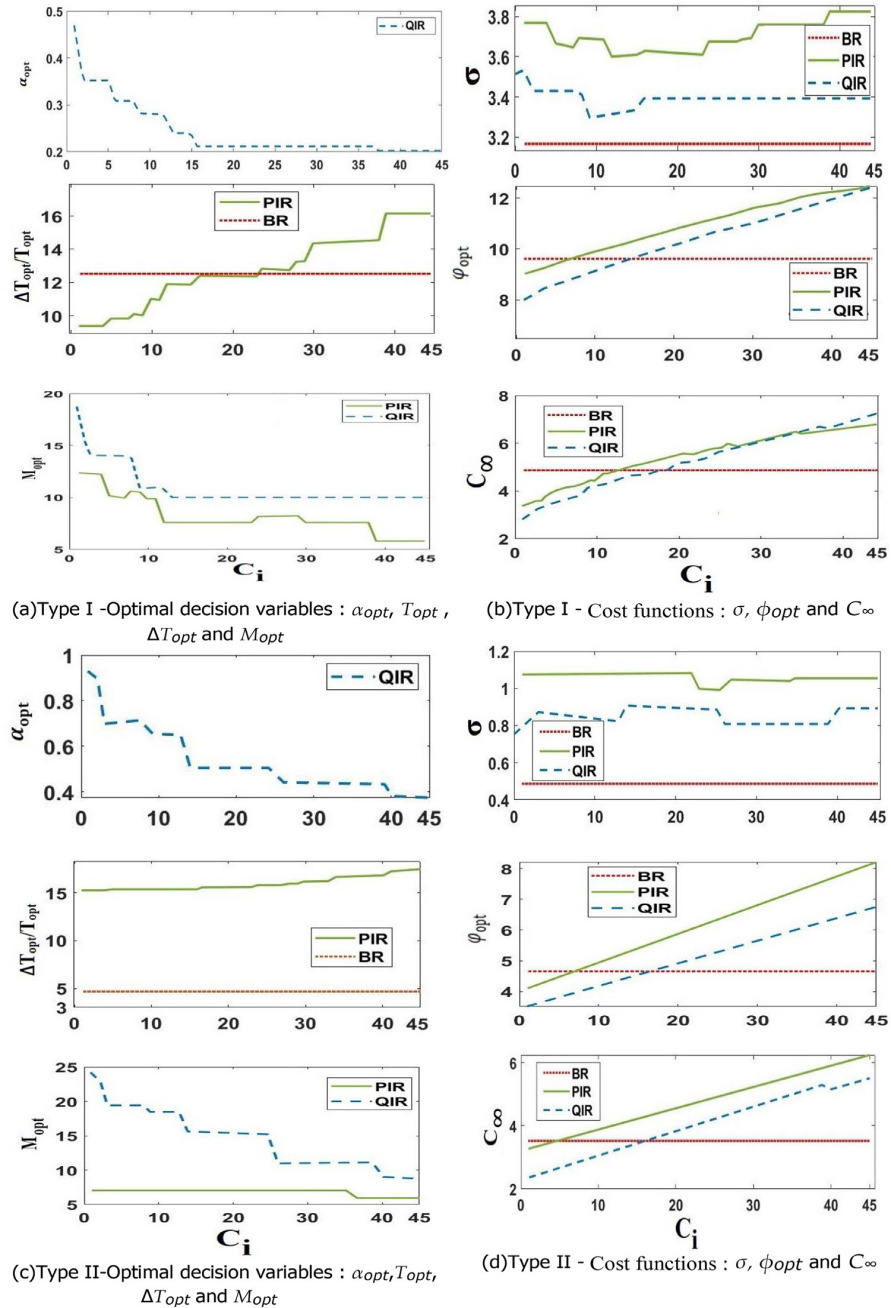
Sensitivity to the inspection cost C_i_.

1) Sensitivity to the Inspection Cost C_i_:

This case study is consistent with the results shown in Fig. 2, where 2a and 2c show the evolution of the best decision variables for Type I and Type II systems, respectively. Fig. 2b and 2d show the corresponding costs for these optimum options in both types of systems. It is anticipated that Topt will remain constant in Fig. 2a and 2c for both types of systems since the BR method functions without the need for inspections. On the other hand, the PIR and QIR approaches depend on inspection costs, and their variables of choice are highly sensitive to changes in those costs.

To be more precise, when inspection costs are large (small values of C_i_), α_opt_ for the QIR strategy and ∆T_opt_ for the PIR approach assume high and low values, respectively, in the case of the system type I with variable behavior (Fig. 2a). This guarantees that the deterioration condition of the system is monitored more often. In the meanwhile, in order to optimize the system's useful life, the M_opt_ decision variable for both methods is set to a high value.

The goal of maintenance techniques is to decrease the frequency of inspections as they become more costly. This is why α_opt_ and ∆T_opt_ have been adjusted to greater and lower values, respectively. When C_i_ reaches extremely high levels, the inter-inspection periods for the PIR and QIR methods may occasionally even exceed T_opt_ of the BR approach. Furthermore, in order to reduce system downtime and avoid excessive inspection expenses, their optimal preventative thresholds (M_opt_) are set to tiny levels, allowing system replacement from the first inspection date.

The QIR and PIR strategies for the Type II system (Fig. 2c) respond similarly to the Type I system in terms of changing decision variables (decreasing α_opt_ and M_opt_, and increasing ∆T_opt_ with the rise in C_i_). The important difference is that when C_i_ is low, Mopt (very close to L) and αopt (very near to 1) assume very high values. By setting M_opt_ to a high value, the QIR approach tends to keep the system running as long as feasible in this case (small values of C_i_).

This setup seems appropriate for a system that exhibits little behavioral variability. Conversely, the system is managed using inter-inspection intervals that are set in accordance with a very high conditional reliability threshold, where αopt is in close proximity to 1. When C_i_ is low (∆T_opt_ ≈ T_opt_), the PIR approach for a stable behavior system (Type II) acts similarly to the BR strategy. But because inspections come at a higher cost, when C_i_ rises, the latter tends to lengthen the time between inspections in an effort to offset the expense increase.

When C_i_ is comparatively low, the PIR and QIR techniques both show great benefits. On the other hand, they are less profitable than BR, particularly when C_i_ is very high (see Fig. 2b and 2d). The reason for this decline in performance is because the PIR and QIR techniques in this case come with an additional expense related to inspection procedures.

Additionally, Fig. 2b and 2d show that for all three techniques, the MCPRC standard deviation is comparatively consistent as C_i_ varies. This implies that variations in the cost of inspection have little effect on how resilient the three techniques are.

It's also important to note that the two conditional maintenance strategies (QIR and PIR) MCPRC standard deviations are somewhat higher than the BR strategy's (with no discernible difference). This suggests that, in general, the decision framework of TBM methods is more resilient than that of conditional maintenance strategies (CBM).

But the latter are excellent at reducing maintenance costs, which maintains their performance edge. It is clear by analyzing the MCPRC standard deviation values (σ scale) for the two types of systems that when the system behaves with minimal unpredictability, maintenance techniques are more resilient (σ does not exceed 1.5). However, the resilience of the maintenance techniques is lost when the system exhibits fluctuating behavior (σ approximates 4).

Determining an adequate maintenance framework thus requires striking a compromise between the resilience and performance of maintenance procedures. It turns out that the objective function φ is a trustworthy signal when making decisions. The PIR and QIR techniques beat the BR approach in terms of the objective function φ when Ci is low, but their advantage decreases when C_i_ is high, as seen in Fig. 2b and 2d.

**Fig. 3:**
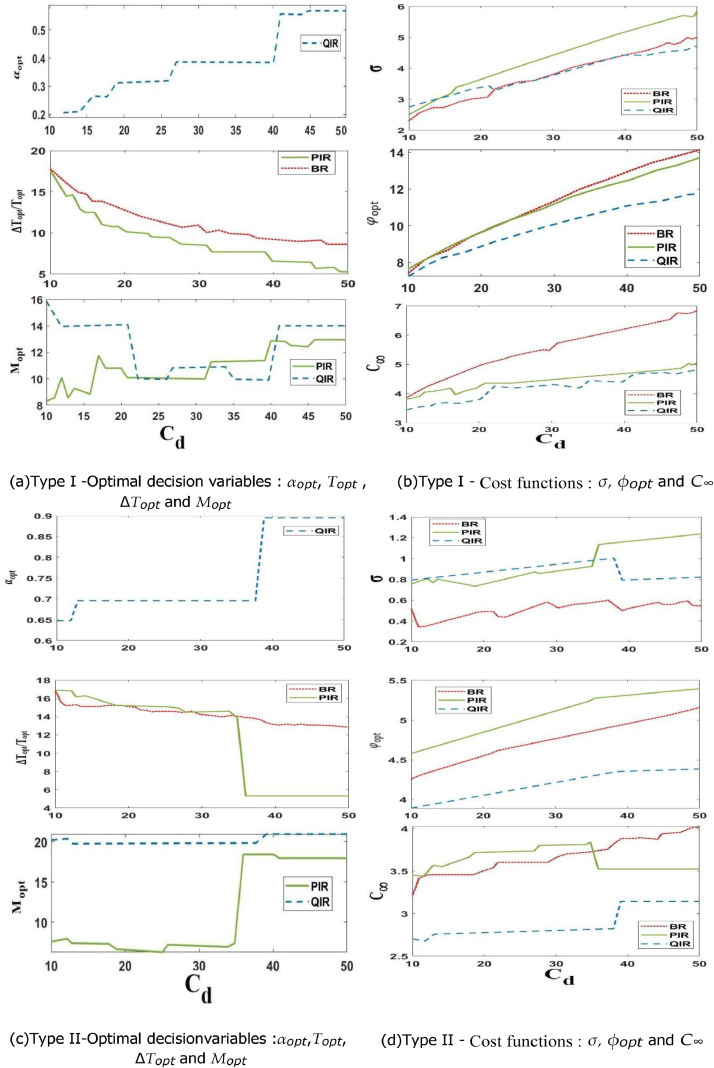
Sensitivity to the system downtime cost rate C_d_.

2) Sensitivity to the system downtime cost rate C_d_:

This study focuses on the second cost configuration that is shown in Fig. 3. We are interested in investigating the impact of the system downtime cost rate C_d_. To be more precise, we just adjust the C_d_ and leave the other unit prices same (as described at the beginning of this paragraph).

The numerical results from this secondary case study are presented in Fig. 3. It is important to note that the interpretations of Fig. 3a and 3b match the ones that were originally provided in the first case study, respectively.

When considering the system with variable behavior (Type I), it is clear that the configuration with C_d_ = 19, which is examined in the second case study, corresponds with the case study scenario where C_i_ = 7. As the system downtime cost rate C_d_ rises, Fig. 3a shows a clear decline in ∆Topt and Topt for the PIR and BR methods, and then a noticeable increase in αopt for the QIR approach.

This pattern of choice variables aims to reduce the significant unavailability costs incurred during such periods by reducing system downtime. The PIR and QIR techniques' more unpredictable preventative threshold (M_opt_) progression is linked to a proportionate cost sensitivity to this parameter.

This explains its supporting role, in which it acts as an extra regulator to help the strategies reach their ideal configurations. Fig. 3c shows that when C_d_ assumes low and moderate values, the inspection intervals of the PIR approach closely resemble the replacement intervals of the BR method for the system exhibiting less erratic behavior (type II).

There is a noticeable change in the way the QIR and PIR methods adapt the decision variables when C_d_ rises to high values, above the C_d_ = 34 threshold. By making this change, they guarantee more regular system monitoring in order to reduce the expenses related to system outages.As a result, the QIR strategy's decision variable α_opt_ quickly becomes near to 1, where as the PIR strategy's a Topt takes a lower value.

A closer look at Fig. 3b reveals that changes in C_d_ have a significant effect on the effectiveness and resilience of the BR, PIR, and QIR methods when it comes to the variable behavior system (type I).

Interestingly, the long-run predicted maintenance cost rate $C_{\infty }$ for the PIR and QIR methods are almost the same, and both are lower than the BR approach (highlighting the value of conditional decision-making). In addition, the PIR strategy's standard deviations are lower and equivalent to those of the BR and QIR strategies for MCPRC, underscoring the advantage of dynamic conditional decision-making CBM.From these results, it can be concluded that, in comparison to the BR and PIR techniques, the QIR strategy performs better and is more resilient.

Within the framework of the Type II system, which is characterized by a standard deviation for MCPRC of this kind of system (Fig. 3d), the strategies are more robust (σ is less than 1.5) than those observed in the Type I system (σ is closer to 7).

As a result, under this scenario, the effect of fluctuation in C_d_ on the resilience of maintenance methods is much reduced (as seen by the comparatively constant standard deviation values for MCPRC). Moreover, Fig. 3d makes it clear that the QIR's cost objective function (φ) outperforms the other cost functions in this case as well. This confirms that the QIR approach constantly achieves the best possible balance between performance and resilience.

### Sensitivity to the relative weight of the cost variability λ

**Fig. 4:**
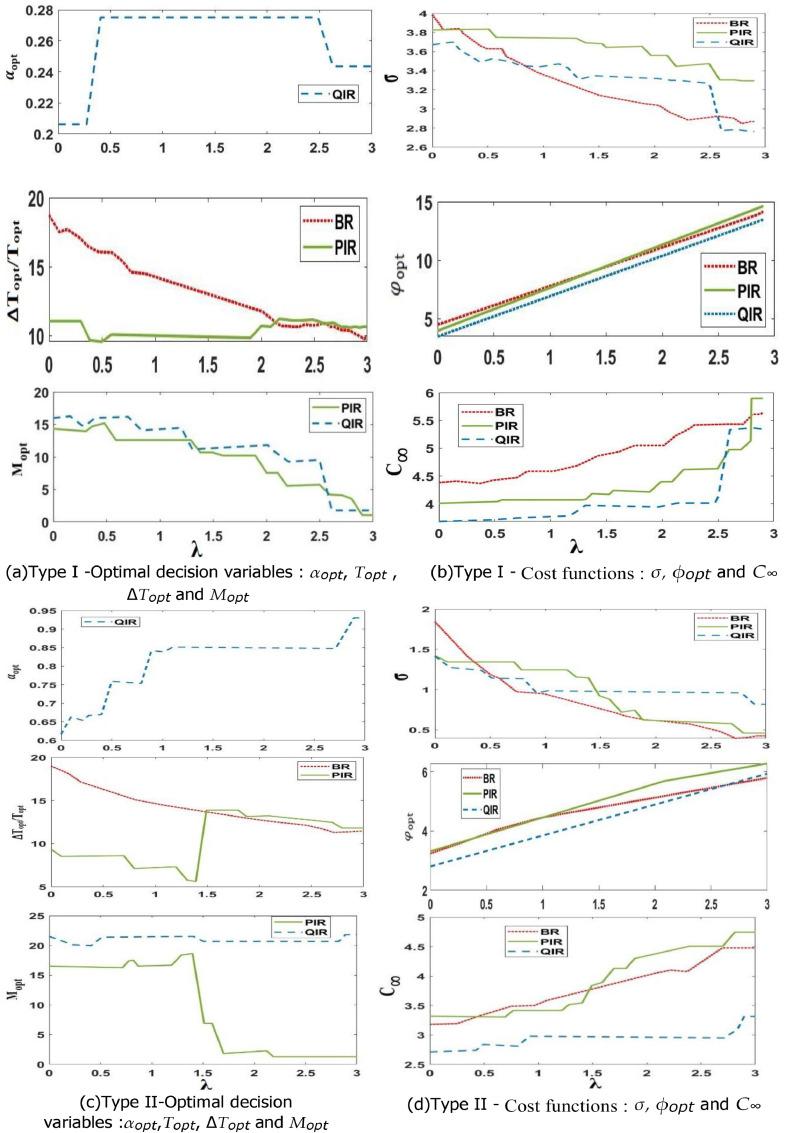
Sensitivity to the relative weight of the cost variability λ.

The proportional weight of the cost variability, or parameter λ, in this study captures the decision-makers' financial hesitancy and risk appetite when choosing a maintenance schedule. Quantifying the effect of λ on the resilience and effectiveness of the planned maintenance techniques is crucial. We set the maintenance costs at C_i_ = 5, C_d_ = 34, C_c_ = 98, and C_0_ = 48 for this reason. Then, we systematically adjust λ in steps of 0.1 from 0 to 3, while monitoring the trajectories of critical decision variables, such as Topt, ∆Topt, αopt, and Mopt. We also examine the relevant cost metrics φ_opt_, C_∞_, and σ for the three maintenance techniques (BR, PIR, and QIR) that are being considered. This thorough process is applied to two different kinds of systems, which are described at the beginning of this chapter. Fig. 4a–d visually represent the results of this investigation.

A purposeful shift in favor of valuing the robustness of maintenance procedures over performance is indicated by the increasing value of λ. Fig. 4a and 4c illustrate a noticeable decrease in Topt, a critical decision variable for the BR method, regardless of whether it is applied to Type I or Type II systems. In fact, the more resilience that is required (as λ increases), the more important it is to prevent system failures. .. For systems displaying varied behavior (type I), we observe in Fig. 4a that ∆T_opt_ and α_opt_ for the PIR and QIR techniques, respectively, stay generally steady. But as λ increases, their preemptive thresholds show a decreasing tendency. This tendency is especially noticeable for variable behavior systems (type I), where conditional maintenance techniques (CBM) offer the greatest advantages. Remarkably, the only choice variables sensitive to changes in the relative weight of the cost variability λ are those that correspond with the conditional aspect (Mopt for the PIR and QIR methods). To achieve the necessary trade-off between performance and resilience in conditional maintenance systems, careful fine-tuning of these variables is sufficient.

In cases when the system exhibits stable behavior (type I), Fig. 4c tells us that when λ rises, the PIR approach changes to mimic a BR strategy (∆T_opt_ ≈ T_opt_). This is a calculated move meant to maintain a same degree of strength. In contrast, the QIR approach significantly increases the values of α_opt_ while utilizing its dynamic conditioning aspect. Additional insights are shown in Figs. 4b and 4d, which show that the standard deviation of the MCPRC σ and the asymptotic average cost per unit of time, C_∞_, for the three techniques show opposite tendencies with respect to the growth of λ. This confirms the fundamental conflict between robustness and performance. In practice, however, designing a maintenance plan that is resilient and performs well seems to be a difficult task. However, when it comes to the final objective function φ_opt_, the QIR method consistently performs better than the BR and PIR techniques. This emphasizes how much better it is at striking a more harmonic balance between toughness and performance.

## Conclusion and perspectives

In conclusion, this paper has introduced a novel evaluation criterion for comparing maintenance strategies, which incorporates both long-term average cost and standard deviation of maintenance cost rate per replacement cycle. By applying this criterion to three maintenance options, we have demonstrated its effectiveness in minimizing risks and costs, thereby facilitating optimal decision-making in maintenance planning.

Our research uniquely contributes to the field by providing a quantitative comparison of the robustness and performance of maintenance options. This allows for a more informed understanding of the trade-offs between resilience and maintenance performance, which has significant implications for maintenance strategy selection.

However, it's important to acknowledge the limitations of our model. While our proposed criterion offers valuable insights, it may not capture all relevant factors influencing maintenance decision-making. Future research could explore additional criteria or refine existing ones to provide a more comprehensive evaluation framework.

Furthermore, our analysis has focused primarily on System Type 1 and System Type 2. Future research could extend this analysis to other system types or consider more complex scenarios to enhance the generalizability of our findings.

Additionally, while our study has highlighted the importance of managing system downtime, further research is needed to develop and evaluate specific techniques for mitigating downtime effectively without compromising performance.

In summary, our research contributes to the understanding of maintenance strategy selection by introducing a novel evaluation criterion and providing insights into the trade-offs between resilience and performance. Future research directions include exploring additional evaluation criteria, extending the analysis to other system types, and developing techniques for managing system downtime more effectively.

To encapsulate, this paper introduces a novel evaluation criterion for comparing maintenance strategies, demonstrating its effectiveness in minimizing risks and costs. While valuable, our criterion may not capture all relevant factors. Future research should explore additional criteria, extend analysis to other system types, and develop techniques for managing system downtime effectively. Addressing these areas will advance maintenance strategy selection and contribute to more robust practices.

## Limitations

while the proposed maintenance plan offers numerous benefits such as enhanced asset reliability and performance, it's important to recognize several limitations. These include resource constraints, complexity, technological dependencies, data availability and quality issues, environmental factors, regulatory compliance requirements, organizational culture, and external dependencies. Organizations should carefully consider these limitations and adapt the maintenance plan accordingly to ensure successful implementation and optimization of asset management processes.

## Ethics statements

The authors declare that our work does not involve any human subjects or animal experiments, nor does it include data collected from social media platforms.

## CRediT authorship contribution statement

**Khamiss Cheikh:** Conceptualization, Methodology, Data curation, Formal analysis, Writing – original draft. **E. L. Mostapha Boudi:** Methodology, Investigation, Formal analysis, Writing – review & editing. **Rabi Rabi:** Supervision, Project administration, Funding acquisition, Writing – review & editing. **Hamza Mokhliss:** Visualization, Software, Validation, Writing – review & editing.

## Declaration of competing interest

The authors declare that they have no known competing financial interests or personal relationships that could have appeared to influence the work reported in this paper.

## Data Availability

No data was used for the research described in the article.
